# Learning to represent visual input

**DOI:** 10.1098/rstb.2009.0200

**Published:** 2010-01-12

**Authors:** Geoffrey E. Hinton

**Affiliations:** Department of Computer Science, University of Toronto, 10 Kings College Road, Toronto M5S 3G4, Canada

**Keywords:** neural networks, learning algorithms, computational neuroscience

## Abstract

One of the central problems in computational neuroscience is to understand how the object-recognition pathway of the cortex learns a deep hierarchy of nonlinear feature detectors. Recent progress in machine learning shows that it is possible to learn deep hierarchies without requiring any labelled data. The feature detectors are learned one layer at a time and the goal of the learning procedure is to form a good generative model of images, not to predict the class of each image. The learning procedure only requires the pairwise correlations between the activations of neuron-like processing units in adjacent layers. The original version of the learning procedure is derived from a quadratic ‘energy’ function but it can be extended to allow third-order, multiplicative interactions in which neurons gate the pairwise interactions between other neurons. A technique for factoring the third-order interactions leads to a learning module that again has a simple learning rule based on pairwise correlations. This module looks remarkably like modules that have been proposed by both biologists trying to explain the responses of neurons and engineers trying to create systems that can recognize objects.

## 1. Introduction

The obvious way to falsify a theory of how the human cortex learns to interpret the visual input is to show that its predictions disagree with experimental data. Deciding what the theory predicts, however, can be difficult. The cortex is an extremely complicated nonlinear system whose behaviour can be changed in unexpected ways by modifying the strengths of the synaptic connections. Detailed computer simulations are therefore required to understand what a synaptic learning rule predicts and the simulations usually show that the synaptic learning rule can be rejected without even considering the experimental data because it simply does not work well enough to have any chance of explaining obvious facts about people's learning abilities. Falsification by simulation has the advantage that it is possible to design better learning rules by analysing how naive learning rules fail. This paper describes a historical sequence of progressively more powerful learning rules that have emerged from computer simulations.

Consider the task of assigning a class label, such as ‘cat’ or ‘dog’, to an image that contains a single salient object. A good way to perform this computation in a network of neuron-like processing elements is to use a hierarchy of progressively more complicated feature detectors (Selfridge 1958; Fukushima 1980; LeCun *et al.* 1998; Serre *et al.* 2007). At each level in the hierarchy, a feature detector is activated by bottom-up input from a particular spatial arrangement of active feature detectors at the level below. Single cell recordings in the visual systems of mammals (Felleman & Van Essen 1991) are consistent with this model and show that the individual feature detectors become progressively more tolerant to variations in retinal position, orientation and scale as we ascend the hierarchy. This raises the question of how such a hierarchy could be learned.

## 2. Learning by back-propagating error derivatives

In the 1980s, the back-propagation algorithm (Werbos 1974; Rumelhart *et al.* 1986) created much excitement because it appeared to solve the problem of learning multiple layers of nonlinear features. Back propagation is a method for computing how to change the connection weights in a feed-forward neural network composed of multiple layers of artificial neurons. An input vector is presented at the bottom layer and in the ‘forward’ pass, each neuron computes a weighted sum of the inputs it receives from the layer below, puts this sum through a smooth nonlinearity and sends the result to neurons in the layer above. The output of each neuron in the final layer is compared with the desired output vector provided by a supervisor and some measure of the discrepancy is used to compute the error for that training case. Each neuron in the final layer, for example, could represent a class and the supervisor could specify which neuron should be active. Derivatives of the error are then propagated backwards through the network using the same connection weights as on the forward pass. Once the error derivatives have been computed for the ‘hidden’ neurons in the intermediate layers, it is straighforward to change their incoming weights in the direction that reduces the error, thus performing gradient descent in the error function.

Back propagation worked impressively well for some problems (LeCun *et al.* 1998), but for many others it did not succeed in using the multiple layers of features to significantly improve performance. In deep nets, the learning was very slow and had a tendency to get stuck in poor local optima. Also, to learn a large number of weights by back-propagating error derivatives requires a huge number of accurately labelled training examples. The idea of learning connection weights by following the gradient of some objective function is very powerful, but classification error is not a good objective function for learning a visual system.

## 3. Unsupervised learning of feature detectors

Back propagation allows the errors in the outputs of a system to drive the search for an appropriate sequence of feed-forward transformations from vectors of pixel intensities to vectors of class probabilities. A very different approach to the learning task emerges if we consider how the training data are actually created. Physical objects give rise to images via a generative process that involves the intrinsic properties of the object, deformation, viewpoint, lighting, occlusion and many other variables (Horn 1977). The generative process is complicated and highly nonlinear but it is not particularly noisy, so the particular combinations of pixel intensities contain a lot of information about their underlying causes. The class of the object is only a tiny fraction of this information and the class is typically much easier to predict from properties such as the three-dimensional shape of the object than from the raw pixel intensities.

With very limited computational resources, it may be sensible to try to perform classification by ignoring all the information in the image that is not directly relevant to the class of the object, but with the huge computational resources of the human visual system it makes much more sense to first recover the underlying causes of the image such as the surface depths, orientations, reflectances and boundaries (Marr 1982). We know that this is possible because human visual perception in a familiar, natural setting almost always returns a good approximation to the true causes of the visual input. Once the underlying causes have been recovered by modelling the complicated, high-bandwidth channel between the underlying causes and the pixel intensities, the class can be recovered by modelling the simpler, low-bandwith channel between the underlying causes and the class label.

There are several advantages to treating the task of object classification as a post-processing step in a system whose main goal is to infer all of the underlying causes that explain how a retinal image was generated. Given *N* equiprobable classes, each labelled image only provides log_2_ *N* bits of constraint on the mapping from images to labels, so to learn a large number of parameters by optimizing discriminative performance requires a very large number of labelled training cases. Each unlabelled image, however, provides a lot of constraint on a generative model, so generative models with a large number of parameters (e.g. 10^8^) can be learned using relatively few training images (e.g. 10^6^) and these training images do not require labels.

Until recently, the types of generative model that could be fitted efficiently to unlabelled i.i.d. data were very restricted. They consisted of mixture models, which treat each data point as a noisy observation of one of a limited number of prototypes, or linear models, such as factor analysis, which treat each data point as a noisy observation of linearly transformed Gaussian noise. Neither of these model classes is appropriate for highly structured images that are generated by multiple latent variables which interact in nonlinear but very regular ways to produce the observed data. Much more powerful models had been suggested (Ackley *et al.* 1985) but the proposed methods for fitting them to data were hopelessly inefficient.

Over the last two decades, the artificial intelligence and statistics communities have made considerable progress in the procedures for fitting complicated, stochastic, generative models to data (Lauritzen & Spiegelhalter 1988; Pearl 1988). These ‘graphical models’ are expressed as graphs in which the nodes represent stochastic variables and the lack of a connection between two nodes represents some type of statistical independence.

Belief nets are a widely used type of graphical model in which the connections are all directed and there are no directed loops (Pearl 1988). Each variable receives directed connections from its ‘parents’ and its probability distribution, when the model is generating data, is determined solely by the combination of values of its parents using a simple parameterized function or table. This makes it easy to generate samples from a belief net using an ‘ancestral pass’. First, the highest level variables are sampled from their prior distributions and then each remaining variable is sampled from the distribution created by the sampled values of its parents.

Assuming that the functions that determine how a variable depends on its parents are known for all of the variables, the *inference* problem is to determine the joint probability distribution for the remaining variables when the values of a subset of the variables are observed. The *parameter learning* problem is to discover how the probability distribution of a variable depends on the values of its parents. This is done by using a set of training cases each of which consists of the observed values of a subset of the variables. A natural way to perform parameter learning is to search for parameters that maximize the probability that the observed data would have been generated by simply running the generative model. This is called ‘maximum-likelihood’ learning.

The generative model underlying factor analysis is a belief net in which there is only one layer of hidden variables and all of the variables are linear with Gaussian noise. This makes inference and maximum likelihood learning fairly straightforward. By removing all noise from the observed variables and using heavy tailed, non-Gaussian noise to drive the hidden variables, we obtain ‘independent components analysis’ (Comon 1994). This generative model is much better than factor analysis at discovering independent causes and is still relatively easy to fit to data, but extending it to models with multiple hidden layers is difficult.

**Figure 1.**
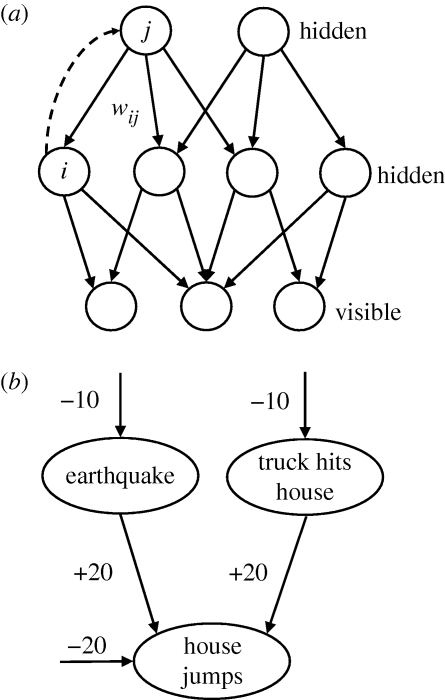
(*a*) A multi-layer sigmoid belief net. The top-down connections define the generative model. Bottom-up connections can be used for inferring hidden states that may have generated the visible data. (*b*) An illustration of ‘explaining away’ in a very simple sigmoid belief net containing two independent, rare, hidden causes that become highly anti-correlated when we observe the house jumping. The bias of −10 on the earthquake unit means that, in the absence of any observation, this unit is *e*^10^ times more likely to be off than on. If the earthquake unit is on and the truck unit is off, the jump unit has a total input of 0 which means that it has an even chance of being on. This is a much better explanation of the observation that the house jumped than the odds of *e*^−20^ which apply if neither of the hidden causes is active. But it is wasteful to turn on both hidden causes to explain the observation because the probability of them both happening is approximately *e*^−20^.

Sigmoid belief nets (Neal 1992) are generative models composed of multiple layers of binary stochastic variables (see figure 1). When the model is generating data, the probability that a variable, *h*_*i*_^*L*^, in layer *L* adopts the value 1 is given by the logistic sigmoid function, 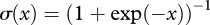
3.1
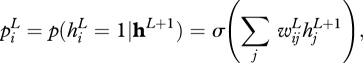
where *w*_*ij*_ is the weight on the connection to unit *i* from unit *j* in the layer above and bias terms have been omitted for simplicity. The binary variables, which will be called ‘units’, can be viewed as crude approximations to cortical pyramidal cells which, over a time period of a few milliseconds, emit a single spike with a probability that depends nonlinearly on the recent input received from other neurons. The lowest level units (those with no descendants) can be used to represent the pre-processed visual input and the higher layers must learn to ‘explain’ each input vector in terms of hidden causes. A good explanation consists of a binary state vector for each layer that is both likely to cause the binary state vector in the layer below and likely to be caused by the binary state vector in the layer above.

Given the parameters of the model, the number of alternative explanations for any given data vector will be exponential in the number of hidden variables, though some explanations will be much better than others. It is infeasible to infer or even to represent the full posterior probability distribution over explanations, but fortunately it is possible to learn the parameters without ever computing the full posterior. It is sufficient to get unbiased binary samples from the posterior. An online, steepest ascent version of maximum-likelihood learning is then3.2
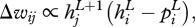
where *h*_*j*_^*L*+1^ and *h*_*i*_^*L*^ are the sampled binary values and *p*_*i*_^*L*^ is the top-down prediction from the sampled values in layer *L* + 1.

Unfortunately, it is hard to even get an unbiased sample from the posterior because of a phenomenon known as ‘explaining away’ that is illustrated in figure 1*b*. Markov chain Monte Carlo methods can be used to get approximate samples from the posterior, but these methods are too slow to be a plausible model of how the brain computes percepts. This leaves two alternatives: use biased samples and hope that the learning procedure still works or find a way to eliminate explaining away so that it is easy to get unbiased samples.

## 4. Learning with incorrect inference

Suppose that instead of sampling a binary explanation, **h**, from the true posterior distribution *p*(**h**|**v**, *W*) given the visible vector **v** and the weights, *W*, we sample from a simpler, approximating distribution *q*(**h**|**v**, *W*) which might, for example, be constrained to be a factorial distribution in which the probabilities of the hidden variables are independent given the data vector, **v**. If we then use the learning rule in equation (3.2), it does not perform gradient ascent in the log probability of generating **v** from a model with weights *W*. It does, however, perform gradient ascent in a closely related quantity called the (negative) variational free energy (Zemel 1994; Neal & Hinton 1998) that differs from the log probability of **v** by the Kullback Liebler divergence between *q*(**h**|**v**, *W*) and *p*(**h**|**v**, *W*)4.1
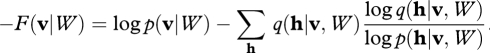


If *q*(**h**|**v**, *W*) = *p*(**h**|**v**, *W*), the variational free energy is minimized, w.r.t. *q*(**h**|**v**, *W*) and performing gradient ascent in −*F*(**v**|*W*) w.r.t. *W* corresponds to maximum-likelihood learning. If *q*(**h**|**v**, *W*)≠ *p*(**h**|**v**, *W*), then −*F*(**v**|*W*) is a lower bound on log *p*(**v**|*W*) and following the gradient of −*F*(**v**|*W*) performs a trade-off between finding parameters that give high probability to **v** and finding parameters that make the approximate inference work well by making the true posterior distribution *p*(**h**|**v**, *W*) close to the approximating distribution *q*(**h**|**v**, *W*). The gradients w.r.t. the parameters of the two terms on the right of equation (4.1) are infeasible to compute because they involve the true posterior, but the gradient of their difference only involves the approximating distribution and is easy to compute if the approximation has a simple form.

Variational learning is now widely used for learning complicated belief nets (Jordan *et al.* 1999). A modified version of variational learning has also been proposed as a model of learning in cortex (Hinton *et al.* 1995). The generative model resides in the top-down connections between cortical areas, and the approximate posterior *q*(**h**|**v**, *W*) is computed by separate bottom-up connections that have their own learning rule. For learning many layers of nonlinear features, however, there is a more efficient method that is based on a scheme for eliminating explaining away so that the posterior distribution really is factorial.

## 5. A stackable learning module

When a multilayer belief net generates data, the stochastic decisions made in one layer influence the probabilities of variables in the layer below, but they have no effect on the probabilities in the layer above because belief nets are ‘directed’ models in which the effects only flow in one direction during the generative process. There is a very different type of model called an ‘undirected’ model in which the effects flow in both directions during generation. An especially simple type of undirected model is a restricted Boltzmann machine (RBM) which contains a layer of binary visible units connected to a layer of binary hidden units, with no connections within each layer. The connections are symmetric, having the same weight in both directions. An RBM is similar to a Hopfield net (Hopfield 1982), but instead of being directly connected the visible units are indirectly connected via the hidden units whose states are not observed. This makes the model more powerful than a Hopfield net because it can use the hidden units to represent higher than the second-order correlations between the visible units, but it also makes the model more difficult to learn.

To generate samples from an RBM, we can alternate between updating all of the hidden units in parallel given the visible states and updating all of the visible units in parallel given the hidden states5.1
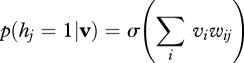
and5.2
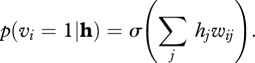


This alternating Markov chain converges to a stationary distribution in which the probability of a joint configuration **v**, **h** of the visible and hidden units is determined by a simple energy function5.3
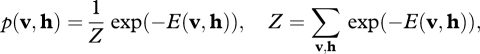
where the energy of a joint configuration is given by5.4
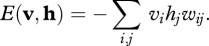


The maximum-likelihood learning rule for an RBM is very simple. Each connection weight, *w*_*ij*_, must be changed in proportion to the difference between two expectations. The first is the expectation that a visible and a hidden unit both have state 1 when the visible vector is sampled from the training data and the hidden states are computed from the visible vector using equation (5.1). The second is the same expectation when the visible and hidden vectors are sampled from the stationary distribution of the Markov chain described above5.5
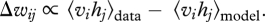


It is conceivable that the second term in equation (5.5) could be estimated by letting an RBM settle to its stationary distribution during an off-line ‘sleep’ phase (Crick & Mitchison 1983) but this would mean that the estimate became progressively worse during the day as the weights changed so it would be hard to learn much in a single day. A more efficient alternative is simply to run the Markov chain for very few steps. First, the visible units are determined by the sensory input and the hidden units are stochastically activated using equation (5.1). Then the visible activities are reconstructed from the hidden states using equation (5.2) and the hidden units are activated again by the reconstruction. This gives a very simple learning rule called ‘contrastive divergence’ (Hinton 2002)5.6
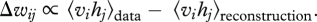


This rule does not maximize the probability that the RBM would generate the training data, but it does work surprisingly well in a wide variety of applications (e.g. Lee *et al.* 2009).

One big advantage of using an RBM, instead of a directed model, is that the hidden units really are conditionally independent given a visible vector, so it is possible to get an unbiased sample from the posterior in one step. This makes perceptual inference accurate, simple and fast. Another big advantage is that RBMs can be stacked to form multi-layer models that are learned one layer at a time. The hidden states of one RBM, when they are being driven by training data, are treated as the ‘data’ for training the next RBM in the stack. The full justification for this recursive learning procedure is given by Hinton *et al.* (2006), who showed how to add extra hidden layers in a way that is guaranteed to improve a variational bound. In other words, the new multi-layer model that is created by adding another hidden layer has a better lower bound on the probability of generating the training data than the previous multi-layer model.

**Figure 2.**
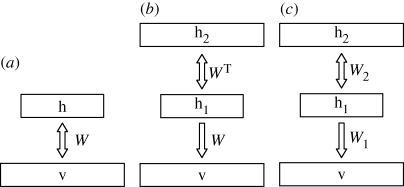
(*a*) A restricted Boltzmann machine (RBM) that consists of two layers of binary variables with undirected connections between layers and no connections within each layer. (*b*) An alternative graphical depiction of the same RBM in which the same weight matrix is used twice. This has no effect on the probability distribution over visible vectors defined by the model. (*c*) A more powerful model produced by allowing the top-level weights to depart from their initial values of *W*_1_^T^ while holding the lower level weights fixed at *W*_1_. The weights *W*_2_ are learned by treating the activity vectors in the first hidden layer as data for training the top-level RBM.

Surprisingly, after training several layers, the composite, multi-layer graphical model is not an undirected multi-layer Boltzmann machine as might be expected, but a ‘deep belief net’ that has an undirected RBM in its top two layers and top-down directed connections from there to lower layers as shown in figure 2*c*. Because of the way in which the multi-layer model is learned, it is possible to use the top-down generative weights in the reverse direction for rapid bottom-up inference, but the bottom-up weights between lower layers are not part of the generative model.

One way to understand the composite model that is created by stacking two RBMs is to write the probability that the first RBM assigns to a visible vector, **v**, in terms of a ‘prior’ distribution over hidden vectors *p*(**h**_1_|*W*_1_) and a conditional distribution of visible vectors given hidden vectors5.7
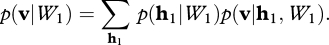


Unlike a normal directed model, the prior uses exactly the same parameters, *W*_1_, as are used for the conditional distribution, *p*(**v**|**h**_1_, *W*_1_). Also, the prior does not assume that the hidden units are independent during the generative process. The prior *p*(**h**_1_|*W*_1_) contains strong correlations that exactly cancel the anti-correlations between hidden units caused by explaining away, so the hidden units are independent in the posterior.

Equation (5.7) makes it clear that the generative model in figure 2*b* is just another way of writing the RBM model in figure 2*a*. But the model in figure 2*b* can now be improved by freezing the bottom layer of weights, *W*_1_, and changing the higher layer of weights to create a better prior distribution for the first hidden layer. A better prior is one that is a better fit to the average, over all training vectors, of the posterior distributions over the first hidden layer given the data. Hence, these posterior distributions over the first hidden layer should be treated as training data for the higher level RBM. After learning a better prior, we have the model in figure 2*c* and the right way to generate data from it is to run a Markov chain to get an unbiased sample of *p*(**h**_1_|*W*_2_) from the top-level RBM and then to do one top-down step to generate a visible vector using *p*(**v**|**h**_1_, *W*_1_).

## 6. Associating feature vectors with labels

Once a high-level representation of the visual input has been learned, there are several ways to associate class labels with that representation. The most obvious method is to treat the high-level representation as the input for a subsequent supervised learning procedure. Excellent performance can be achieved by back propagating derivatives from the supervised procedure through the layers of the deep belief net to refine all of the bottom-up weights that are used for inference. Using back propagation to fine-tune feature detectors that are initially learned as a generative model works much better than using back propagation with random initial weights (Hinton & Salakhutdinov 2006; Erhan *et al.* 2009).

**Figure 3.**
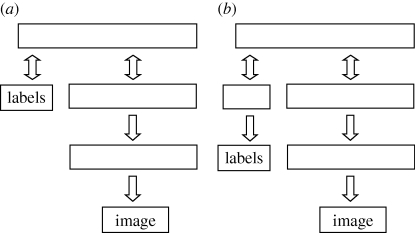
(*a*) After learning two layers of features, a top-level RBM can be trained to model the joint probability distribution of feature vectors and labels. (*b*) If the labels are unreliable, they can be treated as noisy observations of the true labels so that information coming from the image can overrule the label when it is obviously wrong.

An alternative method is to use a top-level RBM to model the joint distribution of feature vectors and labels. The RBM is trained on data obtained by concatenating the high-level representation produced by unsupervised learning with a binary label vector that contains a 1 in the location representing the correct label (see figure 3*a*). To improve discriminative performance, it is possible to add the gradient of the log probability that the RBM would generate the correct label if the feature vector was fixed. This gradient can be computed exactly in a time proportional to the number of possible labels (Larochelle & Bengio 2008).

**Figure 4.**
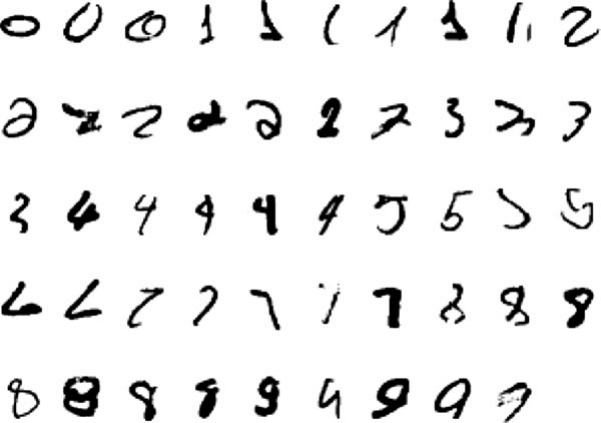
Some test images that the network classifies correctly even though it has never seen them before.

Figure 4 shows what can be achieved by learning the network shown in figure 3*a* one layer at a time using 500 hidden units in the first two hidden layers and 2000 hidden units in the top layer (see Hinton *et al.* 2006 for details).

When many of the labels provided by the supervisor are incorrect, the RBM can use both the provided label and the features obtained from the image to infer the true label. So, if the image is a clear example of some other class, the RBM can overrule the provided label. This works remarkably well and allowed a neural net digit recognizer like the one shown in figure 3*b* to get almost 98 per cent of both the training and the test cases correct even when half of the training labels were wrong. Like a good student, the net ignores the supervisor when the supervisor is obviously wrong but still makes good use of the fact that the supervisor is better than random. This is possible because the unsupervised learning reveals natural classes in the data and the role of the supervisor is primarily to name these natural classes rather than to define them.

## 7. A better module for deep learning

Deep belief nets composed of RBMs are capable of modelling any distribution over binary data vectors even if the width of the hidden layers is constrained (Sutskever & Hinton 2008), but they are a clumsy way to generate some types of structure. Consider, for example, how an officer generates a neat rectangle of soldiers on a parade ground. Instead of directly telling each soldier exactly where to stand, the officer tells them roughly where to stand and also specifies lateral interactions between the soldiers that ensure regular spacing. This greatly reduces the bandwidth of communication required between the officer and the soldiers. Hierarchical systems in which the values of the variables at one level determine the *interactions* between variables at the level below provide a more flexible way to generate or represent complex structures. Each level creates an energy function for the level below, but leaves the details of how to minimize that energy function to the level below, thus avoiding cumbersome micro-management.

RBMs can be modified to allow the states of the hidden units to modulate pairwise interactions between the visible units. The energy function is redefined in terms of three way multiplicative interactions (Sejnowski 1986) between two visible units, *i*, *j*, and one hidden unit *k*7.1
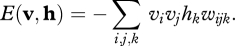


Given the states of the hidden units, the visible units form a Markov random field in which the effective pairwise interaction weight between *i* and *j* is 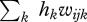. The hidden units remain conditionally independent given the states of the visible units and their binary states are sampled using7.2
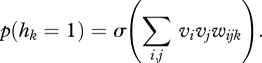


Given the hidden states, however, the visible units are no longer independent so a ‘mean field’ reconstruction of the data from the hidden states is performed by starting at the data vector and using a few iterations of the damped mean field equation7.3
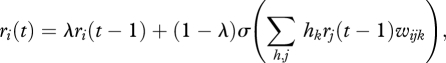
where 0 < *r*_*i*_ (*t*) < 1 is a real-valued approximation to the stochastic binary state *v*_*i*_ and 0 < **λ** < 1 must be large enough to prevent oscillations. After computing the reconstruction, the hidden states are again sampled using *r*_*i*_ and *r*_*j*_ in place of *v*_*i*_ and *v*_*j*_ in equation (7.2). The contrastive divergence learning rule for the third-order weights is then7.4
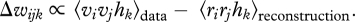


This way of allowing hidden units to modulate interactions between visible units has far too many parameters because each hidden unit has independent control over every pairwise interaction between the visible units. For real images, however, we expect the required lateral interactions to have a lot of regular structure. A hidden unit that represents a vertical occluding edge, for example, needs to modulate the lateral interactions so as to eliminate the horizontal interpolation of intensities in the region of the edge. This regular structure can be approximated by modelling the tensor of three-way weights as a sum of ‘factors’, *f*, each of which is a three-way outer product7.5
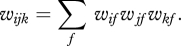


Thus equation (7.1) becomes7.6
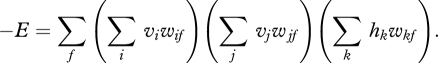


The factors are deterministic and, unlike the stochastic visible and hidden units, they must send different messages to different sets of stochastic units. To sample a hidden unit from its posterior distribution given the states of the visible units, the input to the hidden unit must be the reduction in the energy of the whole system caused by turning the hidden unit on7.7
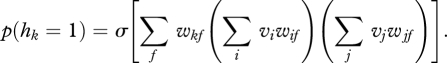


If we assume that the same weights between pixels and factors are used in the first and second weighted sums in equation (7.6), the message that factor *f* must send to every hidden unit is7.8
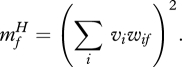


By similar reasoning, the message that factor *f* must send to every visible unit is7.9
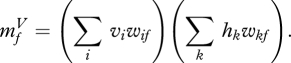


The use of factors that send different messages to different places is a form of belief propagation (Kschischang *et al.* 2001). It ensures that the hidden units remain conditionally independent which is crucial for fast, accurate inference. It also makes contrastive divergence learning very simple. For the factor-to-hidden weights7.10
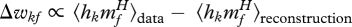
and for the visible-to-factor weights7.11
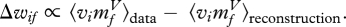


**Figure 5.**
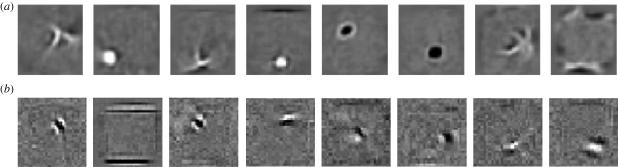
(*a*) Shows the receptive fields of the hidden units of an RBM after training on images of handwritten digits. (*b*) Shows the receptive fields of the three-way factors after training on some lower resolution handwritten digits. Each factor controls an additive contribution to the inverse covariance matrix of the pixels. The strength of the contribution is proportional to the products of the weights from the factor to the two pixels, so the bar-shaped receptive fields contribute strong positive correlations in the direction of a stroke. They also contribute strong negative correlations in the orthogonal direction for pixels separated by about the width of the stroke.

The use of a third-order energy function has led to a module for deep learning containing an intermediate layer of deterministic factors that act as linear filters which send their *squared* outputs to the hidden units via weighted connections. This is exactly the ‘oriented energy’ model (Adelson & Bergen 1985) that is widely used in computer vision. It is also very similar to a standard model of the interaction between simple and complex cells in visual cortex which is supported by both psychophysical evidence and single cell recordings (Carandini *et al.* 2005), though the standard model also includes divisive normalization. By deriving this familiar model from a third-order energy function, we obtain a simple, local learning procedure for all of the parameters. Figure 5 shows how the receptive fields learned by the third-order factors differ from the receptive fields learned by a standard RBM when trained on the images of handwritten digits. The ‘Gaussian scale mixture’ model (Portilla *et al.* 2004) is a closely related, directed graphical model with multiplicative interactions, but inference and learning are more complex because of explaining away.

Third-order, factorized energy functions can also be used to stabilize the interactions between visible units during the reconstruction process. If all of the weights of a three-way factor are constrained to be negative and the states of the visible units are constrained to be positive, a factor that is only connected to visible units will act as a gain control that contributes energy which grows cubically as the activities of the units increase. The message that the three-way factor must send to the visible units is the squared output of a linear filter, as in equation (7.8), so the incoming negative weights can all be implemented by positive weights, but the outgoing weights must remain negative. This resembles an inhibitory interneuron and is one way to implement divisive normalization.

Factorized, third-order RBMs can be stacked to form composite models that have many hidden layers. This work has only just begun, but it should lead to very powerful generative models. If neurons in the first hidden layer represent local oriented energy, neurons in the second hidden layer should represent local spatial distributions of oriented energy. They should therefore resemble SIFT features (Lowe 1999) which were designed by hand but were originally motivated by neurophysiology. SIFT features are widely used in computer vision systems for recognizing objects.

## 8. Conclusion

Learning procedures that are inspired by biology but evaluated by their computational performance have become much more sophisticated over the last few decades. This is leading to a convergence between adaptive computer vision systems and models of the object recognition pathway in the cortex. As computers become more powerful, this trend is likely to continue. Eventually, our understanding of how to engineer adaptive visual systems should become good enough to allow us to hear what the experimental data are telling us about how the cortical visual system is learned.
